# Pre-operative Concomitant Radio-chemotherapy in Bulky Carcinoma of the Cervix: A Single Institution Study

**DOI:** 10.4137/cmo.s489

**Published:** 2008-03-27

**Authors:** Anne de la Rochefordiere, Youlia Kirova, Severine Alran, Corine Plancher, Virginie Fourchotte, Philippe Beuzeboc, Vincent de Margerie, Peter Petrow, Xavier Sastre-Garau, Vincent Servois, Suzy Scholl, Paul Cottu, Laurent Mignot, Patricia de Cremoux, Remy Salmon

**Affiliations:** 1 Radiation Oncology, for Gynaecology Study Group of the Institut Curie, Paris, France; 2 Surgery, for Gynaecology Study Group of the Institut Curie, Paris, France; 3 Biostatistics, for Gynaecology Study Group of the Institut Curie, Paris, France; 4 Medical Oncology, for Gynaecology Study Group of the Institut Curie, Paris, France; 5 Radiology, for Gynaecology Study Group of the Institut Curie, Paris, France; 6 Pathology, for Gynaecology Study Group of the Institut Curie, Paris, France

**Keywords:** cervix cancer, radiotherapy, chemotherapy, brachytherapy, treatment, radical hysterectomy

## Abstract

**Objective:**

To evaluate the treatment results of patients (pts) with FIGO stage IB2, IIA, IIB cervical carcinoma (CC) treated with pre-operative radio-chemotherapy, followed by extended radical hysterectomy.

**Methods:**

Retrospective study of 148 women treated to the Institut Curie for operable FIGO Stage IB2 to IIB, biopsy proved CC. Among them, 70 pts, median age 46 years, were treated using the same regimen associating primary radio-cisplatinum based chemotherapy, intracavitary LDR brachytherapy, followed by extended radical hysterectomy. Kaplan-Meier estimates were used to draw survival curves. Comparisons of survival distribution were assessed by the log-rank test.

**Results:**

Complete histological local-regional response was obtained in 56% of the pts (n = 39). Residual macroscopic or microscopic disease in the cervix was observed in 28 pts (40%). All but one had in-situ microscopic residual CC. Lateral residual disease in the parametria was also present in 9 pts, all with residual CC. Pelvic lymph nodes were free from microscopic disease in 56 pts (80%). Eight of 55 (11%) radiological N0 patients had microscopic nodal involvement, as compared to 6/15 (40%) radiological N1 (p = 0.03). Seventeen pts (25%) had residual cervix disease but negative nodes. After median follow-up of 40 months (range, 8–141), 38/70 patients (54.1%) are still alive and free of disease, 6 (8.6%) alive with disease, and 11 (15.8%) patients were lost for follow-up but free of disease.

**In conclusion:**

The treatment of locally advanced CC needs a new multidisciplinary diagnostic and treatment approach using new therapeutic arms to improve the survival and treatment tolerance among women presenting this disease.

## Introduction

Despite the improvement in management, the survival of advanced stages cervical cancer is still poor (1, 2). Most studies showed that the cervical tumor size is a significant negative prognostic factor and is inversely correlated with both survival and time to recurrence (3). Bulky primary tumours with diameter over 4 cm in patients with International Federation of Gynaecology and Obstetrics (FIGO) stage IB2 to IVa cervical carcinomas are often associated with a higher incidence of nodal metastases and distal recurrences (1–3). Nine randomized trials have demonstrated the superiority of combined chemotherapy with radiation as compared to radiation alone in the treatment of advanced cervix cancer (3–12). The results of these trials demonstrate a consistent improvement in disease-free and overall survival. Therefore, the radiotherapy and platinum based chemotherapy was recognized as standard treatment for advanced cervical cancer (10). However, the treatment protocols of chemotherapy, volumes and doses of radiation therapy, as well as surgical approach varied making difficult the comparison of the studies (3–12).

As from 1991, Institut Curie progressively introduced concurrent platyl-based chemotherapy in the treatment of bulky cervix carcinoma. Treatment policy was also routinely based on lower dose of pre-operative pelvic irradiation, aiming at decreasing the rate of urinary complications following extended radical hysterectomy. This retrospective study was provided to evaluate the treatment results of patients with FIGO stage IB2, IIA, IIB cervical carcinoma treated with pre-operative radio-chemotherapy, intracavitary brachytherapy, followed by extended radical hysterectomy.

## Patients and Methods

### Patients

From August 1991 to August 2005, 148 women, median age 48 years [21–88], were treated to the Institut Curie for operable FIGO Stage IB2 to IIB, biopsy proved carcinoma of the cervix. Among them, 70 patients, median age 46 years (range, 24–80), were treated using the same regimen associating primary radio-chemotherapy, intracavitary low dose rate brachytherapy, followed by extended radical hysterectomy and constitute the study population. Only 28 patients received concurrent chemotherapy and irradiation (RT-CT) before 1999 as compared to 42 patients (60%) as from 1999.

All patients were clinically staged by the surgical oncologist as well as by the radiation oncologist, usually under general anaesthesia. All biopsies were reviewed for histology and viral study. All patients had to be previously untreated and presented histologically confirmed squamous cell carcinoma or adenocarcinoma of the uterine cervix.

Contrast-enhanced computerised tomography (CT) or more recently magnetic resonance imaging (MRI) of the pelvis and abdomen were used to determine the tumor spread and the nodal status. Last years, thoracic CT scan was added to the staging procedure.

All patients, before the treatment, required to meet all of the following laboratory criteria: hemoglobin level >10 g/dL, WBC count >3000/mm^3^, and/or absolute neutrophil count >1500/ mm^3^, platelet count >100,000/mm^3^, serum transaminase levels <60 IU/mL, total bilirubin level <1.5 mg/dL, serum creatinine level <1.5 mg/dL. Also deemed necessary was adequate cardiopulmonary function that could tolerate radical surgery.

### Radiation modalities

Treatment protocol consisted of preoperative radio chemotherapy and intracavitary brachytherapy. Pelvic external beam radiotherapy (EBRT) was delivered using 18 to 20 MV photons and a 4-field box technique, all treated every day. CT scan in treatment position was usually performed and patients underwent conformal RT in most cases. The superior limit was L4-L5, inferior included the vagina. Posterior limit was determined using pre-treatment CT scan. Median planned total dose was 36 Gy in 22 fractions of 1.8 Gy, five days a week, following ICRU recommendations (14). All patients underwent weekly clinics and it was recommended to follow a low-fibers diet, combined with anti-spasmodic medication. Daily nurse care of the perineal skin was performed in the institution for all patients treated after 2000.

### Brachytherapy procedure

Each brachytherapy procedure was performed under general anesthesia. The tumor regression was assessed by gynecologic examination after MRI and/or CT scan realized at the end of chemoradiotherapy. Depending on the length of the uterine cavity found during the brachytherapy procedure together with the topography of vagina and cervix as clinically evaluated, the appropriate size, length of the uterovaginal applicator of Delouche type was chosen as well as adapted sources of ^137^Cesium. After implantation, orthogonal X-ray images were performed with a reconstruction jig. The dose distribution was based on anatomic, clinical and implant parameters. Low dose rate (LDR) brachytherapy was realized in all cases using Curithron machine with 137 Cs sources. The prescription was done to 60 Gy reference volumes. Nominal dose of 30 Gy was delivered in reference volume, following ICRU recommendations (14).

### Chemotherapy

Chemotherapy consisted of 5 Fluorouracil (5FU) 600 mg/m^2^ via continuous infusion and cisplatyl 20 mg/m^2^ from day 1 to day 5, starting concurrently with radiation therapy. The antiemetic regimen consisted of a combination of dexamethazone and 5-hydroxytryptamine-3 antagonist. Two chemotherapy cycles were scheduled at 21 day-intervals. Routine blood cell counts and renal function tests were performed every week. No cytokine for augmenting granocyte colony growth was given.

### Surgery

Six weeks after the completion of brachytherapy, all patients underwent radical hysterectomy, bilateral adnexectomy, resection of a vaginal cuff. The ureters were separated from medial attachments to the peritoneum and the parametrial tissue was cut at the level of the ureters. Bilateral pelvic lymphadenectomy with node sample frozen section was routinely performed. In case of nodal involvement, lymphadenectomy was completed to the ipsilateral common iliac nodes.

### Toxicity

Treatment toxicity was assessed weekly throughout the treatment period and scored according to the World Health Organization criteria. Acute side effects were those appearing during treatment. Late toxicity was the symptoms which appeared 6 months after the completion of the treatment.

### Statistical analysis

Kaplan-Meier estimates were used to draw survival curves. Survival rates are presented with their 95% confidence interval. Comparisons of survival distribution were assessed by the log-rank test. *p-value* of less than 0.05 was considered as statistically significant. Overall survival, relapse-free or distant free survivals were calculated from date of diagnosis to the date of local, regional or distant failure. Prognostic factors for local-regional or distant recurrences were assessed by univariate analysis. Prognostic factors were as follows, age at diagnosis (> = 45 versus >45), menopausal status, hemoglobin (<12 versus >/= 12), tumor size (5 cm vs. >5 cm), and stage (T1B2-IIa versus IIB), histological type (squamous vs. adenocarcinoma) and histological grade (well and moderate versus poor differentiation), viral type (18–45 vs. 16 vs. others), clinical as well as pathologic nodal stage, pathologic response to treatment, chemotherapy doses. Due to the number of the study population, we did not intend to perform multivariate analysis.

## Results

### Patients

The studied population consisted of 70 patients FIGO Stage IB2 to IIB treated between August 1991 and August 2005 primary radio-chemotherapy, intracavitary low dose rate brachytherapy, followed by radical hysterectomy. The median follow-up was 40 months (range 8–141 months).

Clinical and pathologic characteristics are shown in Table 1. Fifty of the patients (71%) were pre-menopausal. Sixty-eight patients (97.1%) presented normal weight and only 2 patients (3%) were obese. Only one patient had a history of diagnosis of previous cancer (breast). Median hemoglobin level at the diagnosis was 13 g/dl (range, 7–15). Fourteen patients (21.5%) presented haemoglobin levels <12 g/dl. Previous cervical Pap-smear test had been performed only in 34 cases. The mean time interval between normal smear and diagnosis of cervical cancer was 18 months.

Eighty three percent of the tumors were squamous cell carcinomas, and 17% were adenocarcinoma. Papilloma virus type (HPV) distribution is shown in Table 1.

Median clinical tumor size was 5 cm (range, 2.9–10 cm). There were 23 (33%) tumor invasions to the upper vagina, and/or 40 (60%) to the proximal parametria. The clinical involvement of parametria was as follows: none in 30 cases (42.9%), unilateral in 32 pts (45.7%), bilateral in 8 cases (11.4%).

The median elapsed time between the first diagnosis et the beginning of treatment was 3 months.

Forty percent of the tumors were clinically Stage IB2-IIa and 60% were FIGO IIB.

Radiological abdominal and pelvic investigations consisted of CT scan in 25 patients (36%), of MRI in 18 patients (26%) and of both exams in 27 patients (38%).

Totally, the imaging procedure depended from the year of treatment, as follows: 71.4% of all patients (50/70) underwent CT scan and 64.2% (45/70)—MRI.

Investigations revealed radiological pelvic nodal involvement in 15 patients (21%). No patient had para-aortic suspicious node. All patients were free from clinical or radiological distant metastasis.

### Radiation therapy

All patients completed the planned radiotherapy.

The median dose delivered to the pelvis was 36 Gy (range, 35–38 Gy) in 20 fractions and 27 days (range 25–32). Intracavitary LDR brachytherapy was performed 10 days after the external beam radiotherapy (range 4–15 days). Nominal dose of 30 Gy was delivered in reference volume. The mean dimensions of the 60 Gy reference volume were H = 10.5 cm, W = 7.5 cm, T = 6.5 cm, combined with external beam radiotherapy. Median rectal and bladder nominal doses received from brachytherapy treatment, at reference points, were 22 Gy (range, 8–36 Gy) and 13 Gy (range, 4–26 Gy), respectively.

### Chemotherapy

All patients received at least one cycle of 5-Fluorouracil-cisplatin chemotherapy. Because of hematological toxicity, 6 patients (8%) interrupted their systemic treatment after one cycle of chemotherapy. Sixty-four (92%) patients received their 2 planned full-dose cycles of chemotherapy.

### Compliance and acute toxicity

Early toxicity was usually mild and reversible (Table 2).

Due to hematological and/or gastrointestinal toxicity, radiation therapy was discontinued in 6 patients. Four of those patients interrupted their treatment for one week and the two other patients for two weeks. Six patients (9%) received only one cycle of chemotherapy. In addition, 5 patients delayed their second cycle of chemotherapy: 4 with 1 week and 1 patient with 2 weeks.

Grade 3 leucopenia and thrombocytopenia were observed in 10% and 13% of the patients, respectively. Two patients presented Grade 4 leucopenia. Four patients underwent blood transfusion before the start of the treatment, but no further patient required transfusion during the combined protocol. All patients complained of severe fatigue (grade 3) during the three first days following the administration of chemotherapy. Patients generally recovered spontaneously after four or five days.

Nausea and vomiting were very moderate: 100% of patients presented grade 1 and 2 nausea and only one patient grade 2 vomiting.

Grade 3 and 4 diarrhea occurred 2 weeks after day 1 of chemo-radiotherapy and was significantly increased after the administration of the 5 FU. Grade 2 gastro-intestinal disorders were observed at the fourth week of radiation therapy.

Due to intensive perineal nurse care, skin reaction was generally limited to grade 1. Two patients only developed grade 3 skin reaction during the treatment.

Neurotoxicity and cardio toxicity occurred only in 1 patient (the same one).

A couple of patients developed grade 1 rectal bleeding after combined chemotherapy and radiotherapy followed by brachytherapy. This symptom disappeared under local corticosteroid therapy. No patient presented any hematuria or fistula.

### Surgery

Surgery was performed 6 weeks (range, 5–11) after the brachytherapy. All patients underwent radical hysterectomy and pelvic external iliac lymphadenectomy. Ovaries had been transposed and preserved in 4 patients. In 8 cases, the frozen section revealed microscopic nodal involvement leading to extend lymphadenectomy. Among them, the lymphadenectomy was performed to ipsilateral common iliac lymph nodes in 3 patients and bilateral common iliac nodes in 5 patients. In this group three patients underwent para-aortic lymphadenectomy. The total mean number of examined lymph nodes was 10 (range 2–31, SD 5.45).

### Pathologic response

Complete histological local-regional response was obtained in 56% of the patients (39 patients).

Residual macroscopic or microscopic disease in the cervix was observed in 28 patients (40%). All but one had *in-situ* microscopic residual carcinoma. Lateral residual disease in the parametria was also present in 9 patients, all with residual carcinoma of the cervix.

All patients but one had negative surgical margins. Pelvic lymph nodes were free from microscopic disease in 56 patients (80%). Eight of 55 (11%) radiological N0 patients had microscopic nodal involvement, as compared to 6/15 (40%) radiological N1 (*p =* 0.03). Seventeen patients (25%) had residual cervix disease but negative nodes.

### Complications

No treatment-related death was observed. Despite anti-thrombosis safety treatment throughout brachytherapy and surgery, vein thrombosis occurred in two patients as well as pulmonary embolism in one patient. Ultrasound of the urinary tract was performed post-operatively in all patients. No ureteral fistulae or bladder complication was observed.

### Late toxicity

Four patients (5.7%) developed intestinal obstruction at 6 months (3 patients) and 96 months after the completion of surgery. Among them, 3 were treated medically and one surgically. In addition, one patient presented grade 3 proctitis. Two (3%) additional patients presented with grade 2 and grade 3 lymphedema of low extremities, respectively. Four cases of vaginal necrosis (6%) occurred within six months following surgery.

Grade 3 pelvic sclerosis occurred in 2 heavy smokers. Twenty percent of the patients complained of mild dyspareunia due to moderate shrinkage of the vaginal wall. When feasible, hormone replacement treatment significantly improved these disorders.

### Survival

After median follow-up of 40 months (range, 8–141), 38/70 patients (54.1%) are still alive and free of disease, 6 (8.6%) alive with disease, and 11 (15.8%) patients were lost for follow-up (median follow-up: 25 months) but free of disease. Fifteen patients (21.5%) died, all but one from cervical cancer. No patient’s death was associated to treatment complication.

**Figure 1:**
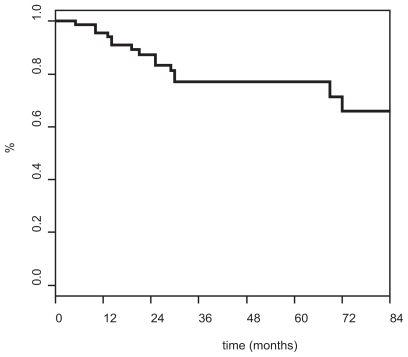
Overall (Kaplan-Meier) survival curve.

**Figure 2:**
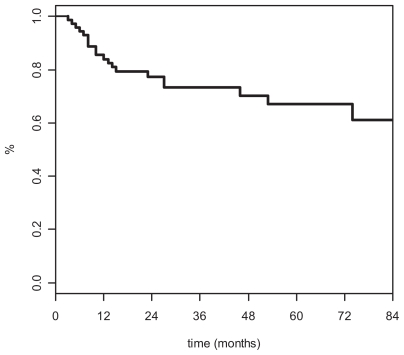
Disease-free (Kaplan-Meier) survival curve.

Actuarial survival (Fig. 1) and disease-free survival (DFS) rates (Fig. 2) were as follows: 77% [95% confidence interval [CI] 66–89] and 74% [95% CI: 63–85] at 3 years, and 77% [95% CI: 66–89] and 68% [95% CI: 55–81] at 5 years, respectively.

### Local-regional failures

Seven local failures occurred (10%), as follows: one patient presented massive pelvic recurrence with invasion of vagina, bladder, rectum, and lateral pelvic wall 6 months after surgery. Another patient recurred in the low third of the vagina, and five patients recurred in the parametria and pelvic lymph nodes within 18 months. Local failures were associated with para-aortic nodal recurrence in two cases and with distant failure in four cases.

Actuarial local and regional recurrence free survival rate was 90.5% [95% CI: 84–98] at 3 years and 87% [95% CI: 78–97] at 5 years.

### Distant failures

Crude rate of para aortic and distant metastases was 25%. Twelve (67%) of the eighteen distant failures were not associated with a local and/or regional recurrence. Para-aortic lymph node failures were documented in 8 patients (11%). Among them, 3 were isolated, 2 were associated with pelvic nodal failure, and 3 with peritoneal recurrence. Five patients were diagnosed with isolated lung metastases.

Actuarial distant free survival rate was as follows 76% [95% CI: 66–87] at 3-years and 69% [95% CI: 57–84] at five years.

### Univariate analysis of prognostic factors for local or distant failure

As previously shown, numerous prognostic factors have been studied using univariate analysis; the results are shown in Table 3. Due to the small numbers of patients, we did not intend to perform to a multivariate analysis.

**Figure 3:**
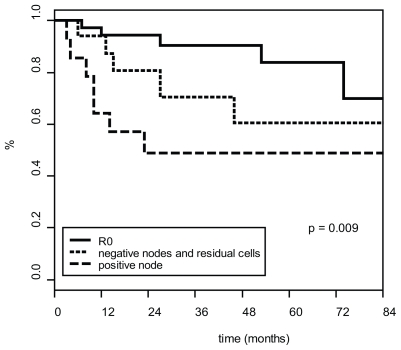
Disease-free survival curve according to pathological T and N response.

In univariate analysis, pathological nodal invasion was the only prognostic factor for survival. The actuarial survival rate at three years was 81% ± 6 in node negative patients as compared to 60% ± 14 in node positive patients ( *p =* 0.05) (Fig. 3). Similarly, radiological or pathological pelvic nodal invasion were significant prognostic factors for local ( *p =* 0.001) and for distant failure ( *p =* 0.001).

Clinical tumor size or stage as well as any residual tumor on surgical specimen did not show any significant influence on local or distant failure rates. This was confirmed by subgroup analyses looking at pathological results in terms of node negative associated or not with residual local tumor. Again, the presence of carcinoma in pelvic nodes remains the only prognostic factor for failure.

## Discussion

The presented experience shows that with respect of Institution’s protocols and the work of an experimented team of specialists, the results in terms of tumor response, DFS, overall survival, and also in toxicity are comparable with the previously published controlled trials. This specific scheme, combining lower dose of EBRT (36 Gy) and higher dose of brachytherapy (30 Gy), combined with surgical resection encompassing the proximal part of the parametrial tissue, shows good loco-regional control and survival, accepted late and early toxicity, and quality of life of our patients. Furthermore, no urinary complication was observed in this study population, whereas other studies reported 9% (6) 15% (15), and 20% (17) of urinary complications with external radiation dose of 45 Gy.

In our study we found 3-years actuarial survival and disease-free survival (DFS) rates respectively of: 77% [95% CI] 66–89] and 74% [95% CI: 63–85]. Corresponding scores at 5 years were 77% [95% CI: 66–89] and 68% [95% CI: 55–81]. These results are similar to the reported results of large controlled studies (7).

Nine reports of randomized trials of concurrent cisplatin-based chemoradiotherapy and one meta-analysis showed that this treatment improves overall survival over various controls in women with locally advanced cervical cancer (3–12). The variation of chemotherapy protocols, different extent of surgical management and radiotherapy modalities among the published trials make interpretation difficult (3–13). Therefore the aim of our study was to report an objectively homogeneous population of patients, treated with the same chemo-and radiation therapy regiment, operated by the same surgeon team, and to try to find any prognostic factors.

Surgical resection allows assessment of pathological response and can improve treatment outcome in the case of a partial pathological response. In the current study, the rate of complete pathological response after chemoradiotherapy (36 Gy) and brachytherapy (30 Gy), as determined based on the analysis of surgically removed tissues, was 56%. Recently, in a multicenter study of surgery after concurrent chemoradiotherapy (45 Gy) and brachytherapy (15 Gy) for the treatment of advanced cervical cancer, a pathological complete response was reported in 39% of patients (67/175) (15). Other studies reported a complete pathological response rates of 52% to 54,2% could be obtained with preoperative chem oradiotherapy (45 Gy, 39.6 Gy) in stage IB2-IIIA cervical cancer (6, 16). Jurado et al. reported a complete response rate of 67.5% (27/40) in a series of patients treated with concurrent chemoradiotherapy (45 Gy), surgery, and intra-operative radiation therapy (17).

Recently, the importance of surgical resection was also shown in the study of the GCCLCC (15). The authors studied 175 patients treated by neoadjuvant chemoradiotherapy, followed by surgical resection. At a median follow-up of 36 months, after hysterectomy, the authors reported an acceptable morbidity and concluded that surgery allows an evaluation of the pathological response to previous treatment and improves local control in the case of partial pathological response (15). In addition, as conclusion of our experience, after radical hysterectomy and pelvic external iliac lymphadenectomy, it is possible in case of positive lymph nodes to add adapted to every case adjuvant treatment. Recently published paper (18) showed 16% lymph node involvement after chemoradiation and concluded that performing a pelvic lymphadenectomy along with the removal of the primary tumor after neoadjuvant treatment could reduce the rate of lateropelvic recurrences. This study supports our treatment strategy of larger surgical resection after chemoradiation therapy. To decrease the side effects after treatment and to approve the quality of life in our patients’ population, this radiochemotherapy regimen proposes a good compromise between the volumes and doses of EBRT and brachytherapy. The adverse effects of this regimen were exclusively transient, comparable with those of other studies. This regimen can be better tolerated because of the only 2 provided cycles of chemotherapy. Kirwan et al. (19) have studied 4580 patients in 19 randomized studies and they showed that more than 1 in 10 patients suffered severe or potentially life-threatening complications in the chemoradiation arms, more than twice as many as in the control group. All factors have been studied: complications of surgery, of radiation therapy (bone marrow included in the radiation field), and side effects of chemotherapy treatment. At the other hand, late toxicity and quality of life are two issues, not always debated in the results of randomized trials. As conclusion, the authors reported that in view of the consistency and extent of the survival benefit for chemo radiation the additional acute toxicity is justified. The authors suggested that all studies should systematically collect chronic toxicity data using a standard data collection form such as the revised common toxicity criteria (19).

This homogeneous single institutional retrospective study showed a high rate of pathologic response and local control as well as no urinary morbidity in patients with advanced cervical undergoing moderate dose of radiotherapy with concomitant chemotherapy followed by radical surgery. It shows also the comparable results with other studies, as well as the advantages of a good multidisciplinary approach in the treatment of cervical cancer. However, this study must be the step to the development of new protocols and techniques in our every day practice. Based on previously published studies, the concurrent chemotherapy is already a routine part of our treatment (20). To improve the treatment tolerance, currently weekly Cisplatin scheme is used, as recommended. In the same time, new treatment modalities are being developed by our team. Based on the recently published data of Bonner et al. (21), a new protocol is currently submitted for discussion, comparing weekly Cisplatin and radiation therapy to Cisplatin plus Cetuximab and radiotherapy. Testing this new approach, we hope to improve the treatment results. Furthermore, the development of new techniques of radiation therapy, as intensity modulating radiation therapy (IMRT), could decrease early and/or late effects of RT. It may also improve the treatment results by means of dose escalation and when needed by including the para-aortic lymph nodes in the treated volume, without increasing the toxicity of this large irradiation field (22). Parallel to these improvement in our treatment protocols, serious work is running in the development of new vaccines against the cervical HPV infection.

The treatment of locally advanced cervical cancer needs a new multidisciplinary diagnostic and treatment approach using new therapeutic arms to improve the survival and treatment tolerance among women presenting this disease.

## Figures and Tables

**Table 1:** Patients’ characteristics (n = 70).

Patients’ characteristics	N° of patients	%
Histology		
- SCC	58	82.9
- Adenocarcinoma	12	17.1
Tumor differentiation		
- Well	42	61
- Moderate	14	19.5
- Poor	14	19.5
FIGO stage		
IB2	19	27.1
IIA	9	12.9
IIB	42	60
N stage (CT scan)		
N0	55	78.6
N1 pelvic	15	21.4
HPV status		
16	38	54.3
18	8	11.4
45	3	4.3
Other (31,33,35,53)	12	17.2
Unknown (Not done)	9	12.8

**Table 2:** Early concurrent chemo-radiotherapy toxicity, (grade 4 is not mentioned in case of no grade 4 toxicity).

Treatment toxicity	Pts	%
**Anemia**		
Grade 0	21	30
Grade 1	30	43
Grade 2	16	23
Grade 3	3	4
**Leucopenia**		
Grade 0	15	20.8
Grade 1	21	30.0
Grade 2	25	36.2
Grade 3	7	10.1
Grade 4	2	2.9
**Thrombopenia**		
Grade 0	30	43
Grade 1	15	21
Grade 2	16	23
Grade 3	9	13
**Abnormal creatinine level**		
Grade 0	68	97.2
Grade 1	1	1.4
Grade 2	0	0
Grade 3	1	1.4
**Weight lost**		
Grade 0	42	60.1
Grade 1	12	17.1
Grade 2	11	15.7
Grade 3	5	7.1
**Skin and mucosal toxicity**		
Grade 0	22	30.2
Grade 1	38	54.6
Grade 2	8	11.4
Grade 3	2	2.9
**Diarrhea**		
Grade 0	15	21.3
Grade 1	31	44.3
Grade 2	13	18.6
Grade 3	9	12.9
Grade 4	2	2.9
**Alopecia**		
Grade 0	57	81.4
Grade 1	11	15.7
Grade 2	2	2.9
No grade 3 and 4		
**Infection**		
Grade 0	59	84.3
Grade 1	3	4.2
Grade 2	4	5.8
Grade 3	3	4.2
Grade 4	1	1.5

**Table 3:** Pre-operative concurrent radiation and chemotherapy: survivals.

Variable	# of patients	3-year survival %	*p-value*	3-year LRFS^1^ %	*p-value*	3-year DRFS^2^ %	*p-value*	3-year DFS^3^ %	*p-value*
**Age at diagnosis**			*NS*		*NS*		*NS*		*NS*
≤45	34	77 ± 8		91 ± 5		78 ± 7		78 ± 7	
>45	36	78 ± 8		89 ± 6		72 ± 9		99 ± 9	
**Menopause**			*NS*		*NS*		*NS*		*0.02*
Yes	20	82 ± 6		94 ± 3		82 ± 6		82 ± 6	
No	50	67 ± 12		87 ± 10		60 ± 12		57 ± 4	
**Hemoglobin**			*NS*		*NS*		*NS*		*NS*
<12	16	81 ± 12		93 ± 7		64 ± 15		64 ± 15	
≥12	54	73 ± 7		89 ± 5		7 ± 6		75 ± 6	
**Clinical T stage**			*NS*				*NS*		*NS*
TIB2	19	83 ± 10		90 ± 7		84 ± 8		84 ± 8	
T2a	9	83 ± 10		100		75 ± 15		78 ± 15	
T2b	42	74 ± 8		89 ± 6		73 ± 7		70 ± 8	
**Clinical T stage**			*NS*		*NS*		*NS*		*NS*
T1B2-T2a	28	83 ± 9		93 ± 5		82 ± 7		82 ± 7	
T2b	42	73 ± 7		89 ± 5		72 ± 7		71 ± 8	
**Tumor size**			*NS*		*NS*		*NS*		*NS*
≤5 cm									
>5 cm									
**Clinical N stage**			*NS*		*NS*		*NS*		*NS*
N0	55	77 ± 6		94 ± 3		77 ± 6		76 ± 6	
N1	15	77 ± 12		79 ± 11		71 ± 12		71 ± 12	
**Histology**			*NS*		*NS*		*NS*		*NS*
Squamous	58	76 ± 5		90 ± 4		76 ± 6		74 ± 6	
Glandular	12	78 ± 14		92 ± 8		75 ± 12		75 ± 12	
**Differentiation**			*NS*		*NS*		*NS*		*NS*
Well	43	79 ± 7		89 ± 5		80 ± 7		80 ± 7	
Mean-poor	27	74 ± 9		93 ± 5		72 ± 9		68 ± 9	
**HPV**^4^			*NS*		*NS*		*NS*		*NS*
Not done	9	75 ± 15		87 ± 12		100		87 ± 12	
18–45	11	58 ± 18		91 ± 9		73 ± 13		73 ± 13	
16	38	80 ± 7		92 ± 5		73 ± 8		73 ± 8	
Other	12	81 ± 12		91 ± 9		75 ± 12		75 ± 12	
**Path. node status**^5^			*0.05*		*0.001*		*0.004*		*0.004*
Negative nodes	56	85 ± 6		98 ± 2		84 ± 5		84 ± 5	
Positive nodes	14	60 ± 14		99 ± 13		49 ± 13		49 ± 13	
**Path. Report**^5^			*NS*		*0.002*		*0.005*		*0.009*
R0^6^	39	86 ± 6		100		90 ± 5		90 ± 5	
negative nodes and residual cells	17	82 ± 12		93 ± 6		71 ± 13		71 ± 13	
Positive nodes	14	60 ± 14		69 ± 13		49 ± 14		49 ± 14	

1 Local recurrence-free survival.

2 Distant recurrence-free survival.

3 Disease-free survival.

4 Papilloma virus type.

5 Pathological.
